# Mutation ∆K281 in *MAPT* causes Pick’s disease

**DOI:** 10.1007/s00401-023-02598-6

**Published:** 2023-06-23

**Authors:** Manuel Schweighauser, Holly J. Garringer, Therése Klingstedt, K. Peter R. Nilsson, Masami Masuda-Suzukake, Jill R. Murrell, Shannon L. Risacher, Ruben Vidal, Sjors H. W. Scheres, Michel Goedert, Bernardino Ghetti, Kathy L. Newell

**Affiliations:** 1 Medical Research Council Laboratory of Molecular Biology, Cambridge, UK; 2 Department of Pathology and Laboratory Medicine, Indiana University School of Medicine, Indianapolis, IN USA; 3 Department of Physics, Chemistry and Biology, Lingköping University, Lingköping, Sweden; 4 Department of Radiology and Imaging Sciences, Indiana University School of Medicine, Indianapolis, IN USA; 5 Present Address: Department of Brain and Neuroscience, Tokyo Metropolitan Institute of Medical Science, Tokyo, Japan; 6 Present Address: Department of Pathology and Laboratory Medicine, Children’s Hospital of the University of Pennsylvania, Philadelphia, PA USA

**Keywords:** Tau, FTDP-17T, *MAPT* mutation ∆K281, Pick’s disease, Silver staining, Luminescent conjugated oligothiophenes, Electron cryo-microscopy

## Abstract

Two siblings with deletion mutation ∆K281 in *MAPT* developed frontotemporal dementia. At autopsy, numerous inclusions of hyperphosphorylated 3R Tau were present in neurons and glial cells of neocortex and some subcortical regions, including hippocampus, caudate/putamen and globus pallidus. The inclusions were argyrophilic with Bodian silver, but not with Gallyas–Braak silver. They were not labelled by an antibody specific for tau phosphorylated at S262 and/or S356. The inclusions were stained by luminescent conjugated oligothiophene HS-84, but not by bTVBT4. Electron cryo-microscopy revealed that the core of tau filaments was made of residues K254-F378 of 3R Tau and was indistinguishable from that of Pick’s disease. We conclude that *MAPT* mutation ∆K281 causes Pick’s disease.

**Supplementary Information:**

The online version contains supplementary material available at 10.1007/s00401-023-02598-6.

## Introduction

Filamentous Tau inclusions are a defining feature of several neurodegenerative diseases, including Alzheimer’s disease (AD), Pick’s disease and progressive supranuclear palsy (PSP) [22]. Six Tau isoforms that range from 352 to 441 amino acids are expressed in adult human brains and are produced by alternative mRNA splicing of *MAPT*, the Tau gene [19]. They differ by the presence or the absence of amino-terminal inserts of 29 and 58 amino acids, and the inclusion of a 31-amino acid repeat encoded by exon 10 in the carboxy-terminal half. Inclusion of exon 10 results in the production of three Tau isoforms with 4 repeats (4R), and its exclusion in another three isoforms with 3 repeats (3R). The repeats and some adjoining sequences constitute the microtubule-binding domains of Tau. They also make up the cores of Tau filaments.

Filaments made of all six Tau isoforms from human brains belong to the Alzheimer fold [14, 16] or the chronic traumatic encephalopathy (CTE) fold [15]. Besides AD, the Alzheimer fold is also found in primary age-related tauopathy, familial British dementia, familial Danish dementia [48] and some prion protein amyloidoses [25]. Besides traumatic encephalopathy syndrome, the CTE fold is characteristic of subacute sclerosing panencephalitis [38] and the Guam and Kii amyotrophic lateral sclerosis/parkinsonism-dementia complex [39]. In PSP, globular glial tauopathy (GGT), corticobasal degeneration (CBD), argyrophilic grain disease (AGD) and age-related Tau astrogliopathy (ARTAG), only 4R Tau isoforms are found in the disease filaments. Different three-layered Tau folds are characteristics of PSP and GGT and distinct four-layered folds are found in CBD and AGD. ARTAG shares a fold with AGD [48]. In Pick’s disease, only 3R Tau is present in the disease filaments, with the Pick fold being extended and two-layered [13].

A link between Tau dysfunction and neurodegeneration was established through the identification of dominantly inherited disease-causing mutations in *MAPT* in frontotemporal dementia and parkinsonism linked to chromosome 17 (FTDP-17T) [28, 36, 51]. Abundant filamentous Tau inclusions are invariably present in brain cells. This work proved that dysfunction of Tau protein is sufficient to cause neurodegeneration and dementia.

Mutations in *MAPT* account for around 5% of cases of frontotemporal dementia (FTD). These mutations are concentrated in exons 9–12, which encode the microtubule-binding repeats of Tau, and in adjacent introns [22]. They have their primary effects at the protein level or affect the alternative splicing of Tau pre-mRNA. *MAPT* mutations that act at the protein level reduce the ability of Tau to interact with microtubules and some mutations also promote the assembly of Tau into filaments. Mutations with a primary effect at the mRNA level are intronic or exonic and most mutations increase exon 10 inclusion. This results in the relative overproduction of 4R Tau and its assembly into filaments. By cryo-EM, Tau filaments from cases with intron 10 mutations + 3 and + 16 share a fold with AGD and ARTAG [40, 48].

Mutation ∆K281 (previously called ∆K280) in exon 10 of *MAPT* reduces exon 10 inclusion by abolishing a splicing enhancer element, resulting in the relative overproduction of wild-type 3R Tau [11]. Two cases with this mutation have been reported. One individual had FTD and abundant Pick bodies made of 3R tau [41, 58], whereas the other had AD with abundant 3R + 4R Tau inclusions and extracellular Aβ deposits, in the absence of FTD [32].

Here we report two previously unpublished siblings with FTD and mutation ∆K281 in *MAPT* who had severe atrophy of the frontal and temporal cortex. Abundant 3R Tau inclusions were present in nerve cells and glial cells. They were labelled by Bodian silver, but not Gallyas–Braak silver or an antibody specific for Tau phosphorylated at S262 and/or S356. The inclusions were labelled by luminescent conjugated oligothiophene (LCO) HS-84, but not by bTVBT4. LCOs are specific amyloid ligands [1]. By cryo-EM, narrow and wide twisted tau filaments were present, with structures identical to those of Tau filaments from Pick’s disease [13].

## Materials and methods

### Family history and clinical evaluation

The mother of both siblings died of cancer, aged 62 years, and their father of diabetic complications and possible cancer, aged 77 years. They were cognitively normal and did not suffer from a neurological or psychiatric disease. They had 13 children, two of whom developed FTD. Genetic analysis was not carried out on the parents or most of the children. A paternal aunt died with a dementing illness in her 80s.

*Case 1.* This Caucasian male was first evaluated aged 50 for a 9-month history of neurological decline (difficulty in completing tasks and problems with word-finding). More subtle behavioural problems may have contributed to the loss of his job as a production supervisor. The medical history included depression, diabetes mellitus and strabism following a cataract operation. Clinical evaluation revealed alexia, apraxia and difficulties with expressing ideas. Emotional blunting, impaired sight and speech problems were in evidence. The patient showed some improvement with donepezil, whilst memantine was without effect. He underwent magnetic resonance imaging (MRI) of the brain twice (5 months apart) on a 1.5 Tesla GE CVi scanner. Over the following 3 years, he developed disinhibition, hyperorality, poor postural control, mutism and left hemiparesis. Behavioural-variant FTD was diagnosed. The patient died aged 54.

*Case 2.* This Caucasian female was first evaluated aged 47 for a fainting spell, following a family report of a 12-month history of progressive memory loss. The medical history included hypothyroidism, hypertension, hyperlipidaemia, cholecystectomy and hysterectomy. She underwent an MRI scan of the brain. Over the following 2 years, the patient was unable to carry out activities of daily living. A neurological examination at age 49 revealed that she had developed alexia and reduced spontaneous speech with perseveration. Her behaviour was characterised by wandering and attempts at eating non-food items. The patient had a Mini-Mental State Exam of 4/30, a Geriatric Depression Scale of 7/30, a Wechsler Memory Scale of 0/50 and a Boston Naming Test of 1/30. The informant FTD battery showed a low capacity for empathy and perspective thinking. The clinical diagnosis was behavioural-variant FTD. The patient died aged 53.

### Genetic analysis

Informed consent was obtained from the next of kin. Genomic DNA was extracted from the frontal cortex of case 1 and a blood sample of case 2. Analysis of *MAPT* exons and corresponding flanking intronic regions was carried out as described [51]. Standard amplification reactions used 50 ng DNA. Reaction products were treated with ExoSAP-IT (USB) and were amplified asymmetrically using DTCS Quick Start kit (Beckman Coulter). The products were analysed on a CEQ 8000 GeXP DNA analysis system (Beckman Coulter). DNA sequences were analysed according to www.ncib.nlm.nih.gov. To confirm deletion of K281 and better visualise the variant allele, exon 10 was amplified, subcloned into pCR2.1 (Invitrogen) and sequenced.

### Recombinant Tau proteins and immunoblotting

Site-directed mutagenesis was used to delete K281 in the 412-amino acid isoform of human Tau (1N4R), expressed from cDNA clone htau46. Wild-type and mutant Tau were expressed in *E. coli* BL21(DE3) and immunoblotted as described [20]. Antibodies BR134, RD3, RD4 and anti-4R were used at 1:1,000. BR134 is a polyclonal antibody that was raised against residues 425–441 of human Tau [19]. RD3 and RD4 are monoclonal antibodies that were made against residues 209–216 from exon 9 of Tau joined to residues 306–313 from exon 11 (RD3) and residues 275–291 from exon 10 of Tau (RD4) [10]. Anti-4R is a polyclonal antibody that was raised against residues 275–291 from exon 10 of Tau with an N279D substitution [8].

### Neuropathology

Brains and spinal cords from cases 1 and 2 were dissected 26 and 24 h after death, respectively. The right hemibrains were divided into coronal slabs that were stored at − 80 °C and the left hemibrains were fixed in 10% buffered formalin. Right hemibrains were used for filament extraction. Tissue sections (8–10 μm) were obtained from formalin-fixed, paraffin-embedded tissues of left hemibrains and stained with haematoxylin–eosin, Luxol Fast Blue with haematoxylin–eosin, Bodian silver and Gallyas–Braak silver. For immunohistochemistry, the following antibodies were used: AT8 (pS202 and pT205 in Tau; 1:300, Thermo Fisher Scientific) [21, 31]; 12E8 (pS262 and/or pS356 in Tau, 1:1,000, Prothena Biosciences) [47]; AT100 (pT212, pS214 and pT217 in Tau; 1:200, Thermo Fisher Scientific) [31, 60]; RD3 (1:3,000, EMD Millipore); RD4 (1:100, EMD Millipore); anti-4R (1:300, gift from M. Hasegawa); LB509 (α-synuclein, 1:100, Santa Cruz); anti-TDP-43 (1:800, Cosmo Bio); 2F12 (Aβ, 1:1,000, Janssen); and glial fibrillary acidic protein (GFAP, 1:100, Dako). Histology and immunohistochemistry were carried out as described [52].

### LCO staining

Frontal cortex from case 1 with mutation ∆K281 in *MAPT*, a case of AD and a case of Pick’s disease, was used. The case of AD has been described (number 2 in [14]), as has that of Pick’s disease (number 8 in [13]). For double-labelling with anti-Tau antibody, frozen sections (10 µm) were fixed in ethanol for 3 min at 4 °C. They were then incubated in water for 4 min, PBS with 2.7 mM KCl for 10 min and PBS with 5% goat serum and 0.1% Triton X-100 for 1 h. Anti-Tau antibody AT8 (1:500, Thermo Fisher Scientific) was added overnight at 4 °C, followed by secondary antibody conjugated with Alexa Fluor 488 or Alexa Fluor 647 (Thermo Fisher Scientific) for 2 h at room temperature. After washing in PBS, brain sections were incubated with bTVBT4 or HS-84 (100 nM) for 30 min at room temperature [49, 50]. They were then washed 3 times with PBS and mounted with Dako mounting medium for fluorescence (Agilent). For double-labelling with LCOs, frozen sections were fixed in ethanol for 10 min at 4 °C, rehydrated and incubated in PBS for 10 min. bTVBT4 and HS-84 (100 nM) were added for 30 min at room temperature. The sections were then washed 3 times in PBS and mounted. They were analysed using an inverted Zeiss LSM 780 laser scanning confocal microscope, with an excitation of 458 nm for HS-84 and 561 nm for bTVBT4.

### Filament extraction

Sarkosyl-insoluble material was extracted from temporal cortex (grey matter) of case 1 and frontal and temporal lobes (grey and white matter) of case 2, as described [55]. The tissues were homogenised in 20 volumes of buffer A (10 mM Tris–HCl, pH 7.5, 0.8 M NaCl, 10% sucrose and 1 mM EGTA), brought to 2% sarkosyl and incubated for 30 min at 37 °C. The samples were centrifuged at 10,000*g* for 10 min, followed by spinning of the supernatants at 100,000*g* for 25 min. The pellets were resuspended in 700 µl/g extraction buffer and centrifuged at 5000*g* for 5 min. The supernatants were diluted threefold in 50 mM Tris–HCl, pH 7.4, containing 0.15 M NaCl, 10% sucrose and 0.2% sarkosyl, and spun at 166,000*g* for 30 min. The pellets were resuspended in 50 µl/g 20 mM Tris–HCl, pH 7.4, 100 mM NaCl.

### Western blotting and immunogold negative stain electron microscopy

Western blotting and immunogold negative stain electron microscopy were carried out as described [20]. For Western blotting, samples were run on 10% Tris–glycine gels (Novex), and the primary antibodies diluted in PBS plus 0.1% Tween 20 and 1% bovine serum albumin. BR133 (N-terminus of Tau), BR134 (C-terminus of Tau) and BR135 (repeat 3 of Tau) have been described [19]. The following dilutions were used: BR133, BR134, BR135 and RD3 (1:4,000), Anti-4R (1:2,000), AT8 (1:1,000) and 12E8 (1:100,000). For immunogold electron microscopy, BR133, BR134, BR135 and AT8 were used at 1:50; MC1, a conformational antibody that recognises a discontinuous epitope (residues 7–9 and 313–322) in Tau [29], was used at 1:10.

### Electron cryo-microscopy

Three μl of the sarkosyl-insoluble fractions were applied to glow-discharged (Edwards S150B) holey carbon grids (Quantifoil Au R1.2/1.3, 300 mesh) that were plunge-frozen in liquid ethane using a Vitrobot Mark IV (Thermo Fisher Scientific) at 100% humidity and 4 °C. Cryo-EM images were acquired using EPU software on a Titan Krios microscope (Thermo Fisher Scientific) operated at 300 kV. For grey matter of case 1 and white matter of case 2, movies were acquired on a Falcon-4 detector at a total dose of 30 e^−^Å^−2^ and a pixel size of 0.824 Å. For grey matter of case 2, images were acquired on a Gatan K3 detector using a pixel size of 0.93 Å. A quantum energy filter with a slit width of 20 eV was used to remove inelastically scattered electrons. See Supplementary Table 1 for further details.

### Helical reconstruction

Datasets were processed in RELION using standard helical reconstruction [26]. Movie frames were gain-corrected, aligned and dose-weighted using RELION’s own motion correction programme [61]. Contrast transfer function (CTF) parameters were estimated using CTFFIND4-1 [42]. Filaments were picked manually. The NPF map from Pick’s disease (EMD-0077 [13]), low-pass-filtered to 20 Å, was used as initial model for the first auto-refinement of datasets. In addition, for the dataset of case 2, the WPF map from Pick’s disease was used as an initial model for the filaments comprising two protofilaments (EMD-0078 [13]). Three-dimensional auto-refinements were performed with optimisation of the helical twist and rise parameters once the resolutions extended beyond 4.7 Å. To improve resolution, Bayesian polishing and CTF refinement were used [62]. Final maps were sharpened using standard post-processing procedures in RELION, and resolution estimates were calculated based on the Fourier shell correlation (FSC) between two independently refined half-maps at 0.143 (Supplementary Fig. 1) [44].

### Model building and refinement

The atomic model of the Pick filament structure (PDB:6GX5 [13]) was docked manually in the density using Coot [12]. Model refinements were performed using *Servalcat* [59] and REFMAC5 [33, 34]. Models were validated with MolProbity [7]. Figures were prepared with ChimeraX [35] and PyMOL [45].

## Results

### Genetic analysis

**Fig. 1:**
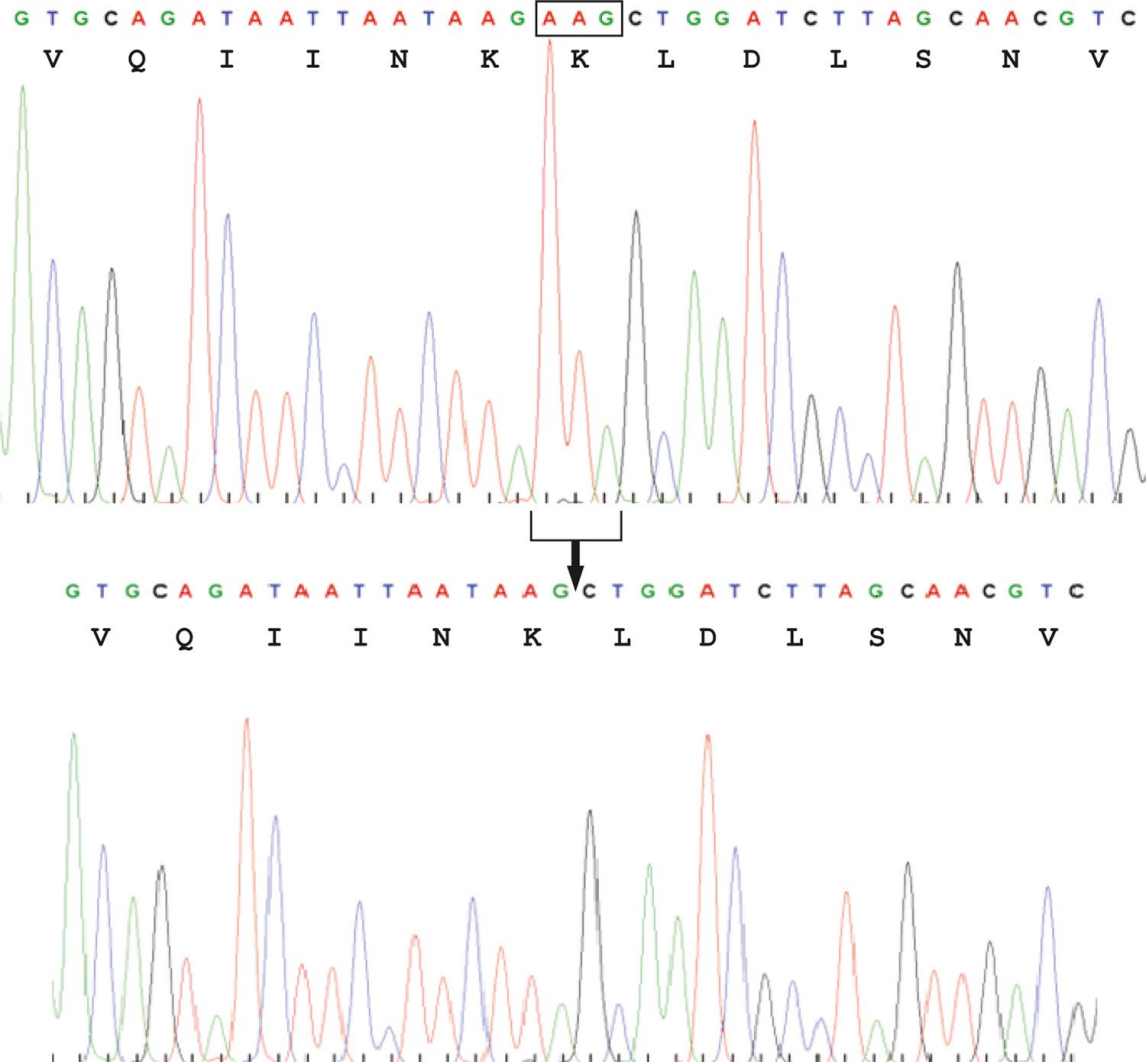
Mutation *∆K281* in *MAPT*. A three-base deletion was present by DNA sequencing following PCR amplification of exon 10 of *MAPT* from case 1. Cloning of the *MAPT* exon 10 amplicon into pCR2.1, followed by sequencing, gave the wild-type sequence in one allele (top) and the deletion mutation in the other (bottom). Identical findings were obtained for case 2. In the wild-type sequence, two consecutive AAG codons encode K280 and K281. Deletion of one AAG codon does not allow one to decide which residue has been deleted. Current convention dictates to name the mutation after the most C-terminal amino acid. We therefore talk of ∆K281

A three-nucleotide (AAG) deletion was present in one allele of exon 10 of *MAPT* in genomic DNA from cases 1 and 2 (Fig. 1). This mutation is also referred to as NM_005910.5:c.841_843del AAG, K281del in NCBI Clin Var [https://www.ncbi.nlm.nih.gov/clinvar/variation/98213/?new_evidence=true]. It was previously called ∆K280 [32, 41, 58]. Residues K280 and K281 are each encoded by AAG. Deletion of the first AAG, AGA, GAA or the second AAG gives rise to what we observed. It is thus not possible to determine which of K280 or K281 was deleted. Current nomenclature guidelines recommend to name such a variant after deletion of the most C-terminal amino acid (http://varnomen.hgvs.org/). It should therefore be called *MAPT* mutation ∆K281, caused by c.841_843delAAG.

### Recombinant ∆K281 4R Tau

Purified recombinant wild-type and mutant ∆K281 1N4R Tau were run on SDS-PAGE and immunoblotted with anti-Tau antibodies BR134, RD3, RD4 and anti-4R (Supplementary Fig. 2). BR134 labelled both wild-type and mutant 4R Tau. RD3 did not label either protein, whereas RD4 labelled wild-type, but not ∆K281 4R Tau. By contrast, anti-4R Tau antibody, which recognises Tau deamidated at N279, labelled both wild-type and ∆K281 4R Tau. Anti-4R was therefore used to detect ∆K281 4R Tau.

### Neuroradiology

**Fig. 2:**
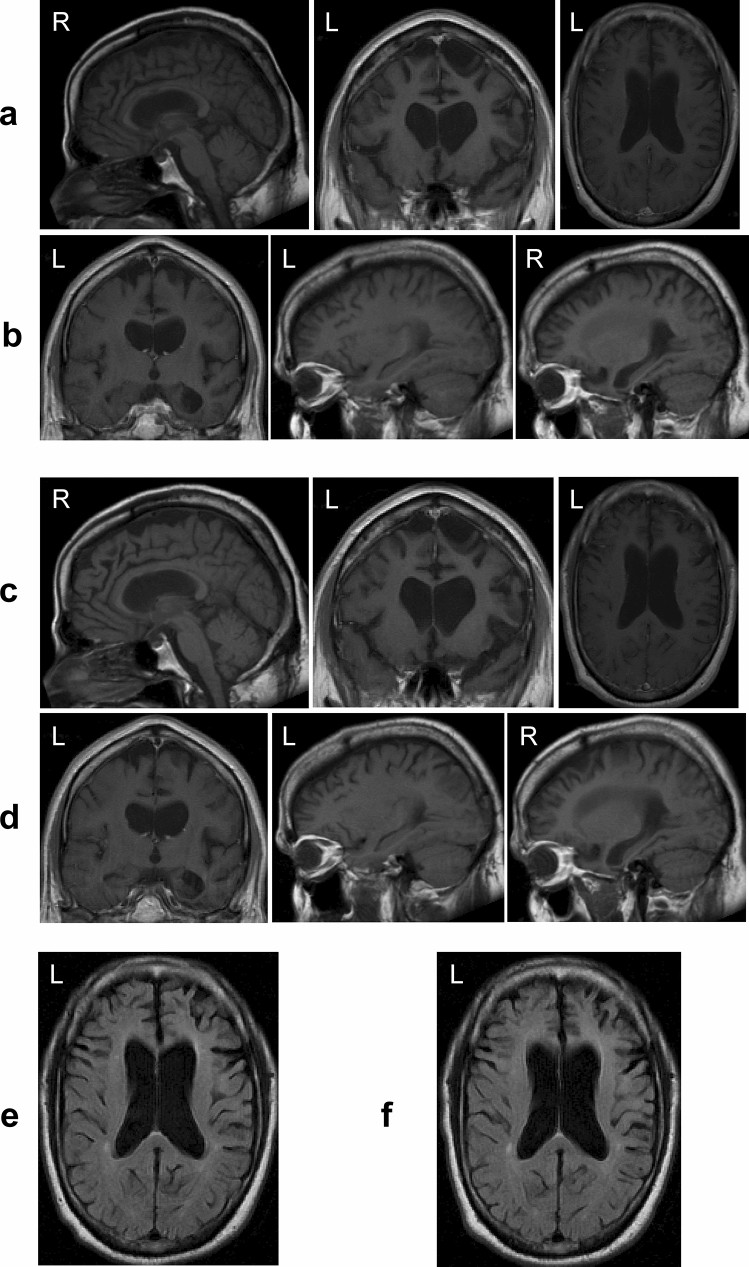
Comparison of two T1-weighted MRI scans of the brain of case 1 with *MAPT* mutation ∆K281. (**a**, **b**, **e**), Scan obtained during the first visit, when case 1 was 50 years old; (**c**, **d**, **f**), Scan obtained 5 months later. (**a**, **c**, **e**), Sagittal, coronal and axial MR images show atrophy of the frontal lobe with knife-blade atrophy of the gyri [middle panel in (**a**) and (**c**)], atrophy of the corpus callosum and severe enlargement of the lateral ventricles. (**b**, **d**, **f**), Coronal and parasagittal MR images show atrophy of the frontal, parietal and temporal lobes, as well as severe enlargement of the lateral ventricles. The MR images from the second visit reveal progression of the atrophy; differences in the dimensions of the lateral ventricles between first and second scans are particularly noticeable

At the time of the first neurological evaluation at age 50, an MRI scan of the brain of case 1 showed pronounced frontal lobe atrophy, mild temporal lobe atrophy, relative sparing of the occipital lobe, atrophy of caudate nucleus and corpus callosum, mild atrophy of the midbrain and cerebellar vermis, as well as severe ventricular enlargement. Atrophy was most severe in the right hemisphere (Fig. 2a, b). A focal area of marked atrophy in the right frontal lobe was associated with the accumulation of cerebrospinal fluid. A second MRI scan was carried out 5 months later (Fig. 2c, d). It showed enlarged sulci and ventricles, as well as the areas of marked atrophy. Mild periventricular white matter signal abnormalities were present, consistent with microvascular ischaemia. An MRI scan of the brain of case 2, carried out at the time of the first neurological evaluation at age 47, showed severe frontal lobe atrophy, mild to moderate temporal lobe atrophy, relative sparing of the occipital lobe, atrophy of caudate nucleus and corpus callosum, mild atrophy of the midbrain and cerebellar vermis, as well as severe ventricular enlargement. Atrophy was most pronounced in the left hemisphere (Supplementary Fig. 3).

### Neuropathology

The cerebral hemispheres of cases 1 and 2 with *MAPT* mutation ∆K281 were atrophic, with frontal and temporal lobes being most severely affected (Supplementary Fig. 4). Parietal and occipital lobes were moderately atrophic. The hemispheric white matter was reduced in volume and the corpus callosum was atrophic. Atrophy of the right cerebral hemisphere was most pronounced for case 1 and that of the left hemisphere for case 2. In both cases, caudate/putamen, amygdala and hippocampal formation were severely atrophic, whereas globus pallidus, thalamus, midbrain and pons were more mildly affected. Substantia nigra and locus coeruleus were mildly depigmented. Nerve cell loss was severe in frontal, temporal, cingulate and insular lobes, as well as in caudate/putamen and globus pallidus. Astrocytic gliosis was severe when nerve cell loss and spongiosis were present. The substantia nigra showed extensive nerve cell loss, with neuromelanin in macrophages and the extracellular space. A moderate loss of cerebellar Purkinje cells was noted.

**Fig. 3:**
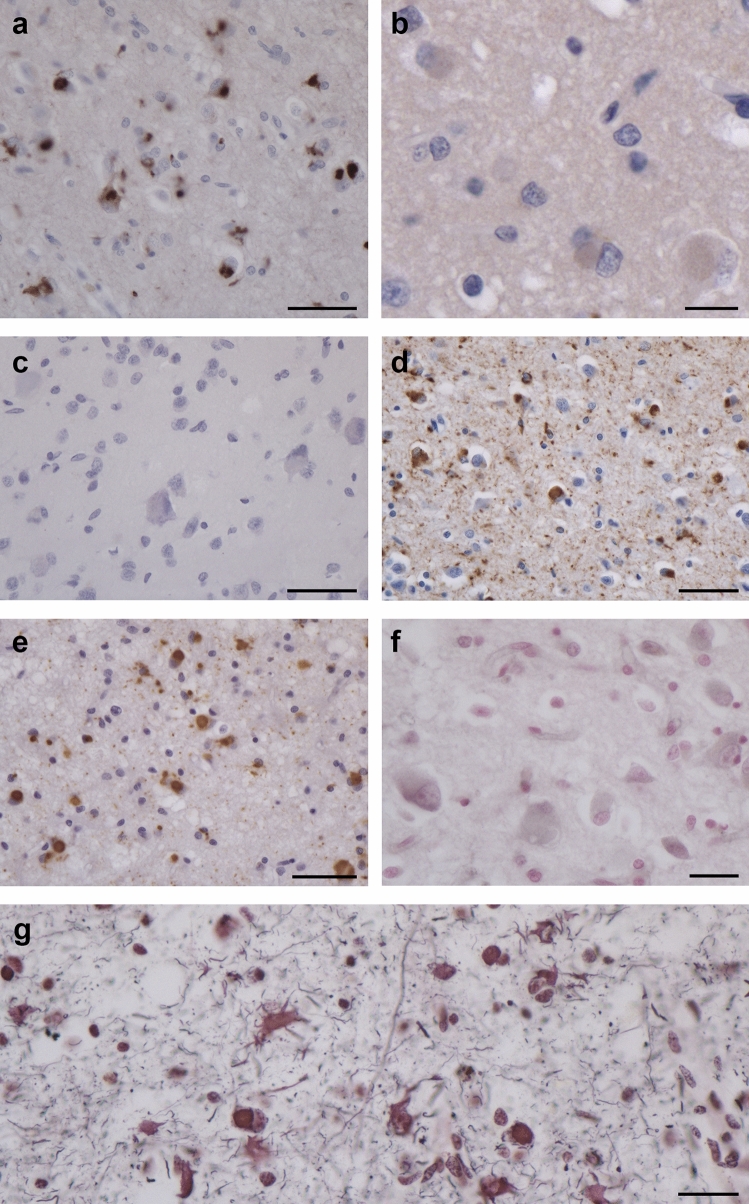
Staining of the frontal cortex from case 1 with *MAPT* mutation ∆K281. (**a**, **d**, **e**), Tau-positive inclusions in nerve cells and glial cells with antibodies RD3 (**a**), AT8 (**d**) and AT100 (**e**). (**b**, **c**), Tau-negative inclusions with antibodies anti-4R (**b**) and 12E8 (**c**). (**f**, **g**), Staining with Gallyas–Braak silver (**f**) and Bodian silver (**g**). Inclusions are not stained by Gallyas–Braak silver, but neuronal and glial inclusions are Bodian silver-positive. Scale bars: 50 µm (**a**, **c**, **d**, **e**), 20 µm (**b**) and 25 µm (**f**, **g**)

**Fig. 4:**
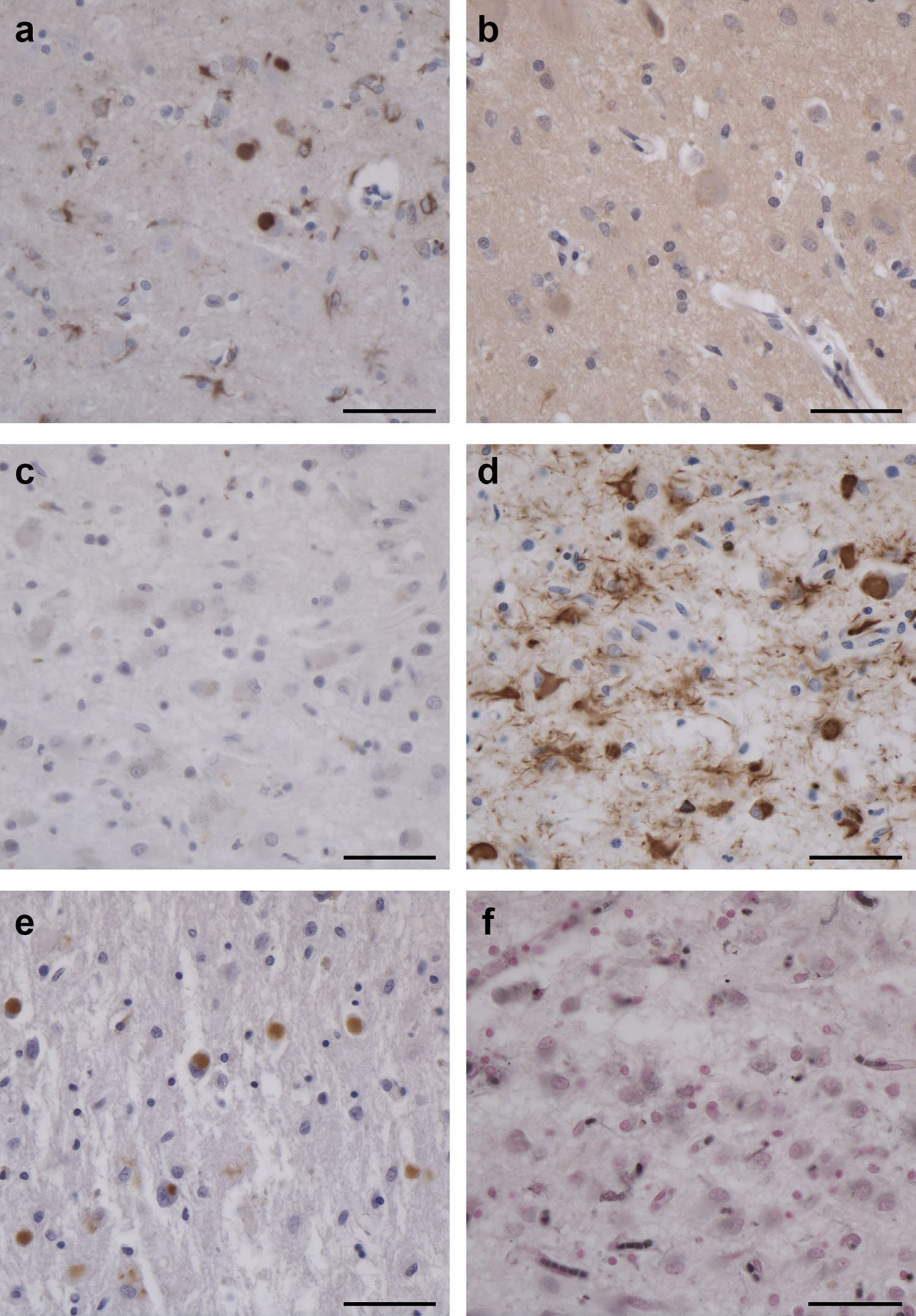
Staining of the frontal cortex from case 2 with *MAPT* mutation ∆K281. (**a**, **d**, **e**), Tau-positive inclusions in nerve cells and glial cells with antibodies RD3 (**a**), AT8 (**d**) and AT100 (**e**). (**b**, **c**), Tau-negative inclusions with antibodies anti-4R (**b**) and 12E8 (**c**). (**f**), Inclusions are not stained by Gallyas–Braak silver. Scale bars: 50 µm (**a**–**f**)

By immunohistochemistry with anti-Tau antibodies, numerous Pick bodies and other inclusions were labelled by RD3, AT8 and AT100, but not by anti-4R or 12E8 (Figs. 3 and 4). They were most numerous in the second, fifth and sixth layers of frontal, temporal, cingulate and insular cortex, as well in dentate gyrus and pyramidal cell layers of the hippocampus. In nerve cells of cerebral cortex and subcortical nuclei that were free of Pick bodies, amorphous tau-positive deposits were present. Anti-Tau antibodies also labelled inclusions in the cytoplasm of astrocytes and oligodendrocytes. Numerous astrocytes were ramified; oligodendrocytes had round or oval inclusions. Ramified astrocytes and oligodendrocyte inclusions were labelled by RD3 and AT8, but not by anti-4R (Supplementary Fig. 5). The cervical spinal cord from case 1 showed numerous tau inclusions. Anti-Aβ antibodies stained a few diffuse plaques in the cerebral cortex from case 1. No staining was observed with antibodies for α-synuclein or TDP-43. Granulovacuolar degeneration was severe in layer CA1 of the hippocampus from both cases. In silver preparations, numerous Pick bodies and other inclusions were labelled with Bodian, but not with Gallyas–Braak silver.

Severe loss of myelinated nerve fibres was present in white matter of the frontal lobe, with a diffuse loss of myelinated fibres in subcortical white matter of the centrum semiovale, as well as in internal, external and extreme capsules (Supplementary Fig. 5). In midbrain, a severe loss of myelinated fibres was present at the level of the frontopontine tract. A good correlation between neuronal loss and pTau immunoreactivity, as detected by antibody AT8, was present in brain and spinal cord of case 1 (Table 1).

**Table 1:** Semi-quantitative neuronal loss and phosphotau (pTau) immunoreactivity

Brain Area	Neuronal loss	pTau	
		Neurons	Glia
Superior/middle frontal gyrus	4	4	4
Superior temporal gyrus	1	0–3	0–2
Middle temporal gyrus	2–3	3	3
Inferior parietal lobule	2–3	1–3	1–3
Occipital cortex	0	2	2
Cingulate gyrus, anterior	4	4	4
Insular cortex	3	3	3
Hippocampus CA1-4	1	1–4	1
Dentate gyrus	1	4	1
Entorhinal cortex	3	3–4	3–4
Amygdala	3	4	3–4
Caudate nucleus	3	2	2
Putamen	3	4	4
Thalamus	1–2	1–2	1–2
Cerebellar cortex	1	0	0
Cerebellum, dentate nucleus	1	1	0
Midbrain, substantia nigra	2	3	2–3
Pons, locus coeruleus	1	4	1
Pons, basis pontis nuclei	1	2	2
Medulla, arcuate nucleus	2	2	1
Medulla, inferior olivary nucleus	1	0	0
Dorsal medulla	0	2	1
Cervical spinal cord	0	2	2

Case 1 was used. None = 0; mild = 1; moderate = 2; frequent = 3; very frequent = 4

**Fig. 5:**
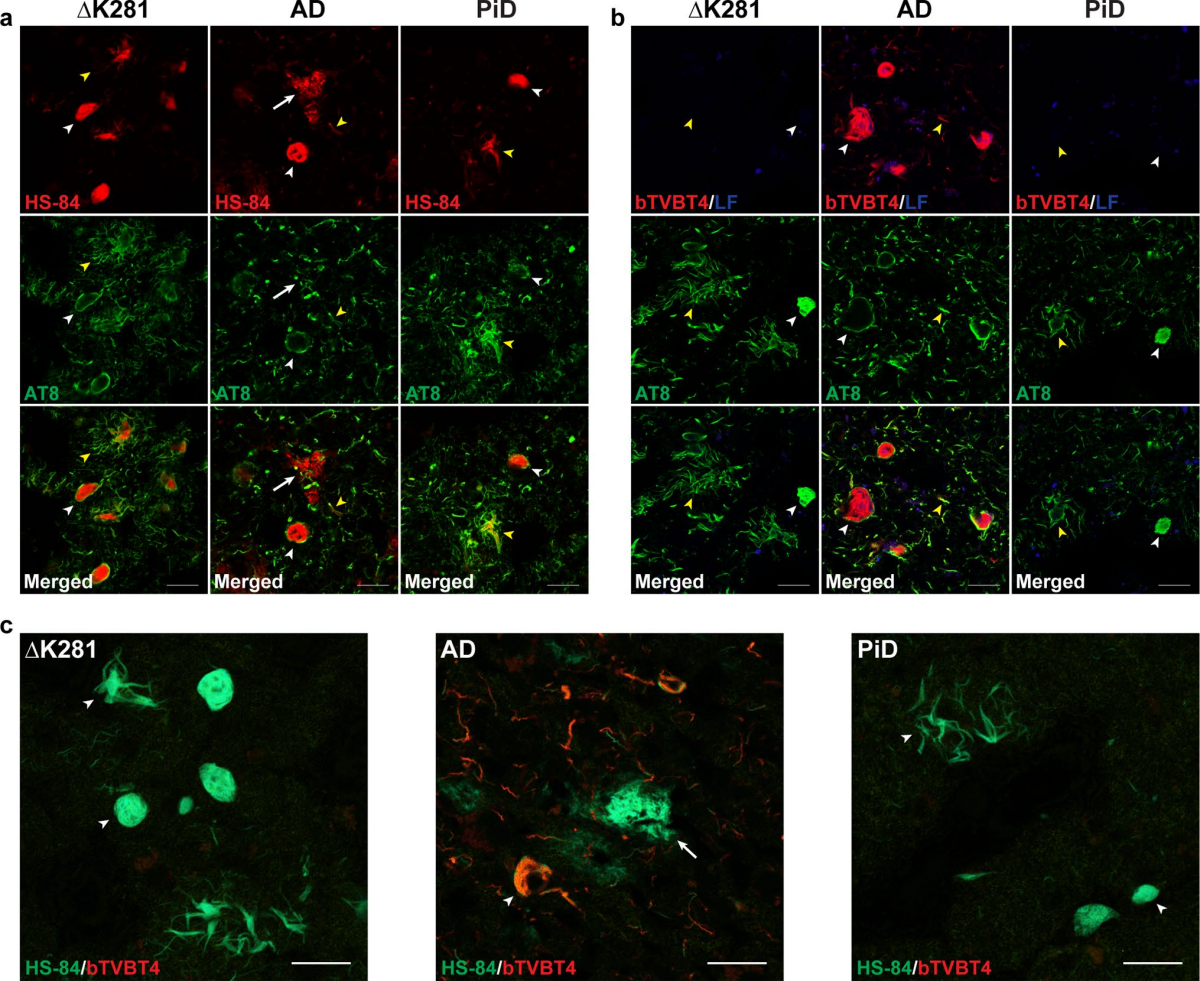
Labelling of Tau amyloid in frontal cortex from case 1 with *MAPT* mutation ∆K281 by HS-84, but not bTVBT4. Comparison with Alzheimer’s and Pick’s diseases. (**a**), Fluorescence images of frontal cortex sections from case 1 with *MAPT* mutation ∆K281, a case of Alzheimer’s disease (AD) and a case of Pick’s disease (PiD) labelled by HS-84 (red) and anti-tau antibody AT8 (green). HS-84 showed co-localisation with AT8 in neuronal (white arrowheads) and glial inclusions (yellow arrowheads) in case 1 (left panel) and in Pick’s disease (right panel). HS-84 showed co-localisation with AT8 in neurofibrillary tangles (white arrowhead) and neuropil threads (yellow arrowhead) in AD (middle panel). HS-84 also identified neuritic Aβ plaques in AD (white arrow). Scale bars, 20 µm. (**b**), Fluorescence images of frontal cortex sections from case 1 with *MAPT* mutation ∆K281, a case of AD and a case of Pick’s disease labelled by bTVBT4 (red) and anti-tau antibody AT8 (green). bTVBT4 did not label neuronal (white arrowhead) or glial tau inclusions (yellow arrowhead) in case 1 (left panel) or Pick’s disease (right panel). bTVBT4 labelled immunopositive neurofibrillary tangles (white arrowhead) and neuropil threads (yellow arrowhead) in AD (middle panel). Blue structures represent autofluorescent lipofuscin (LF). Scale bars, 20 µm. (**c**), Spectral images of frontal cortex sections from case 1 with *MAPT* mutation ∆K281 (left panel), a case of AD (middle panel) and a case of Pick’s disease (right panel) stained with HS-84 (green) and bTVBT4 (red). HS-84, but not bTVBT4, labelled inclusions (arrowheads) in case 1 (left panel) and Pick’s disease (right panel). Both HS-84 and bTVBT4 labelled Tau inclusions (arrowhead) in AD (middle panel). Only HS-84 identified neuritic Aβ plaques (arrow) in AD. Scale bars, 20 µm

When incubated with ligands HS-84 and bTVBT4, case 1 with *MAPT* mutation ∆K281 behaved in the same way as a case of Pick’s disease (Fig. 5). HS-84 labelled Tau inclusions of AD, Pick’s disease and case 1, whereas bTVBT4 only labelled Tau inclusions of AD. Double-labelling with anti-Tau antibody AT8 showed that HS-84 and bTVBT4 stained cell bodies more strongly, whereas AT8 gave stronger labelling of abnormal neurites. This may reflect the denser packing of tau filaments in cell body inclusions and a better penetration by LCOs than by antibodies following ethanol fixation of fresh/frozen tissues. When formalin was used as the tissue fixative, AT8 also strongly labelled Pick bodies.

### Western blotting

**Fig. 6:**
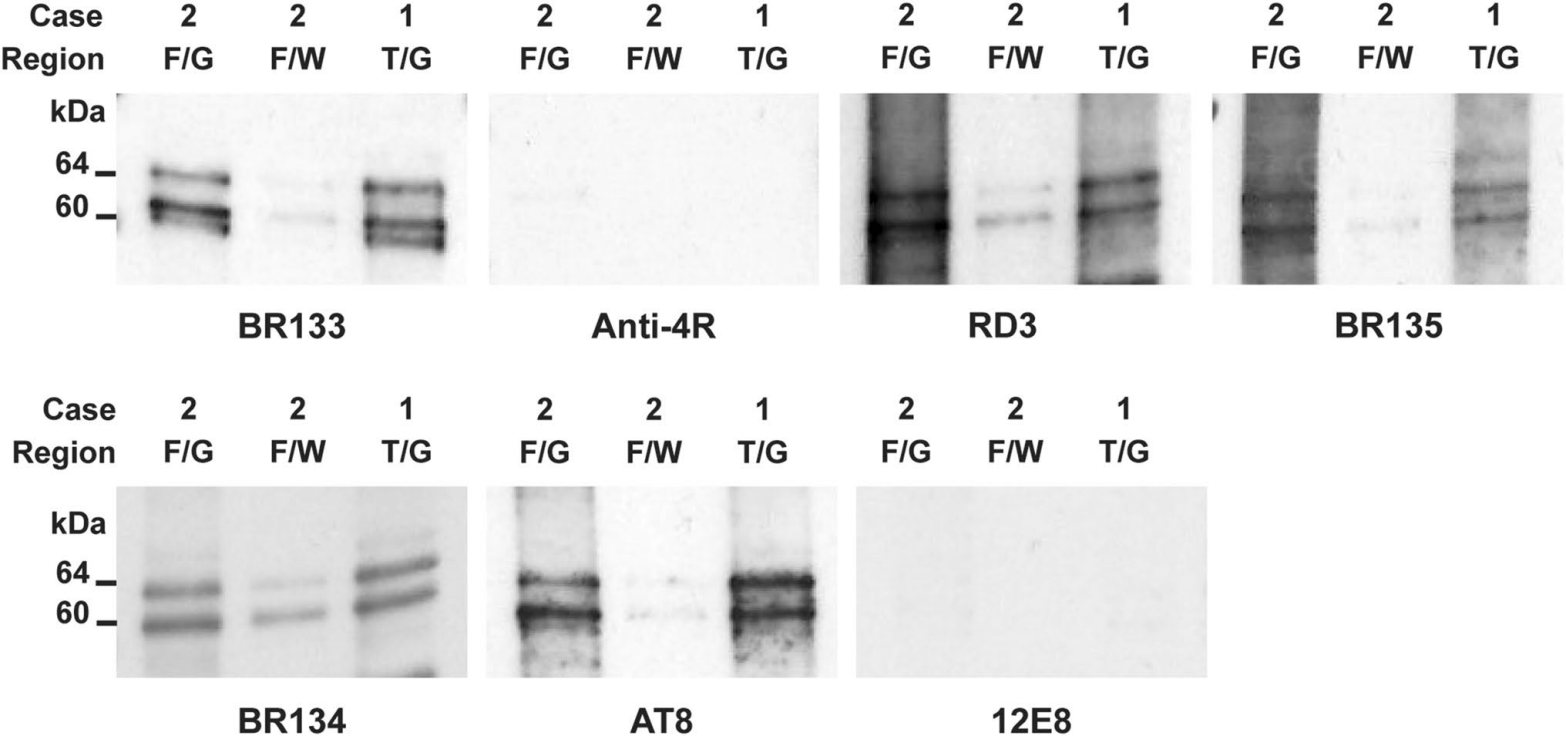
Western blotting of sarkosyl-insoluble fractions from the temproal (T) and frontal (F) cortex of cases 1 and 2 with *MAPT* mutation ∆K281. Two major bands of 60 and 64 kDa and a minor band of 68 kDa were present in grey matter (G). Weaker bands of the same sizes were present in white matter from frontal cortex of case 2 (W). These bands were labelled by anti-Tau antibodies BR133, RD3, BR135, BR134 and AT8. They were not labelled by anti-4R or 12E8

Sarkosyl-insoluble Tau extracted from temporal and frontal cortex of cases 1 and 2 with *MAPT* mutation ∆K281 ran as two strong bands of 60 and 64 kDa, and a weak band of 68 kDa (Fig. 6). They were detected by anti-Tau antibodies BR133, RD3, BR135, BR134 and AT8, but not by anti-4R or 12E8, consistent with the presence of hyperphosphorylated and assembled full-length 3R Tau that was not phosphorylated at S262 and/or S356. Grey matter from temporal (case 1) and frontal (case 2) cortex showed strong Tau-positive bands. They were much weaker in white matter (case 2).

### Electron cryo-microscopy

**Fig. 7:**
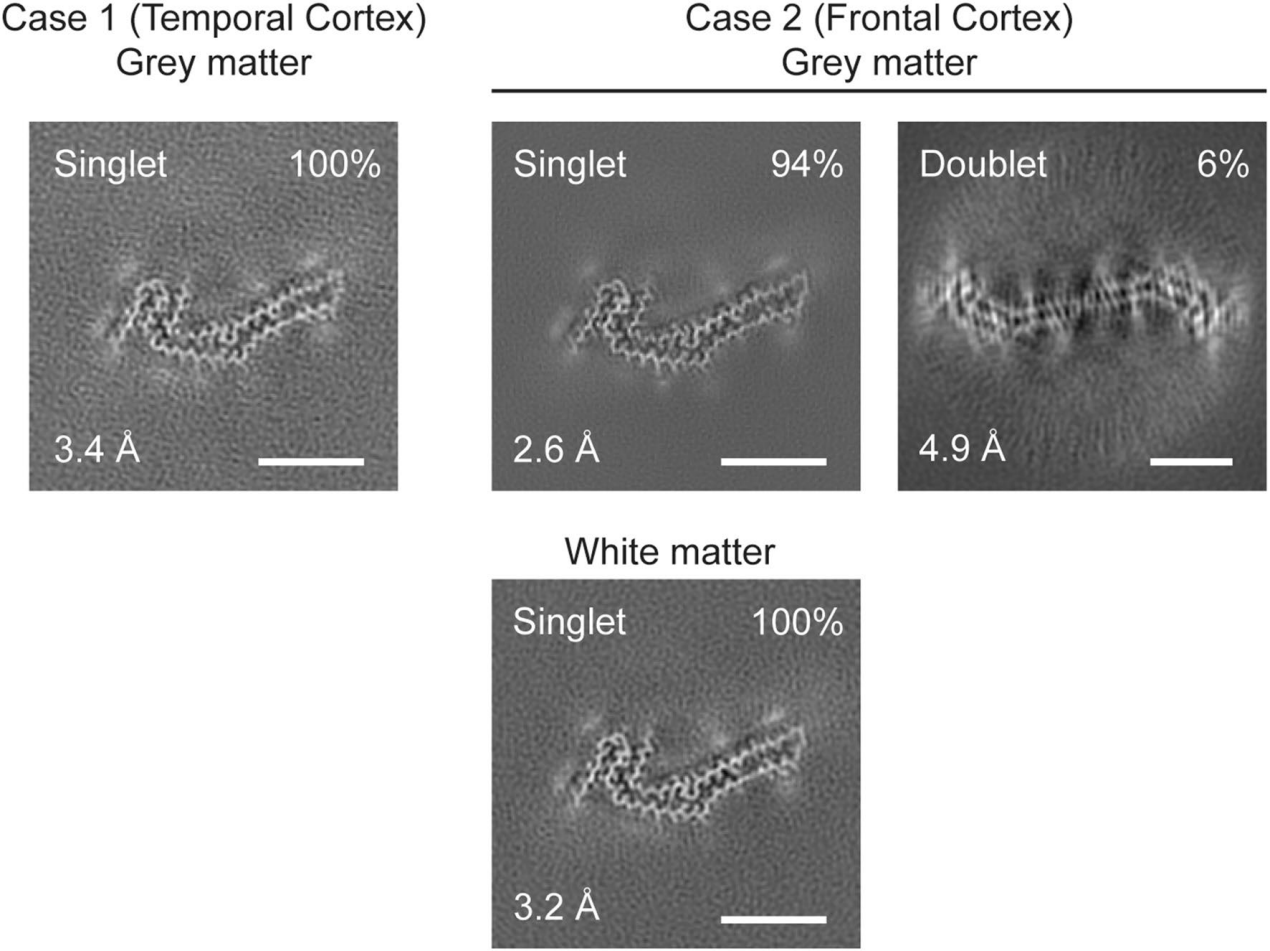
Cryo-EM cross sections of Tau filaments from grey and white matter of frontal and temporal cortex of cases with *MAPT* mutation ∆K281. Cross-sections through the cryo-EM reconstructions, perpendicular to the helical axis and with a projected thickness of approximately one rung, are shown for grey matter of temporal cortex from case 1 and grey and white matter of frontal cortex from case 2 with *MAPT* mutation ∆K281. The resolution of the reconstructions and the percentages of each filament type are indicated. Singlet filaments predominated, with a small percentage of doublets in case 2. Scale bars, 5 nm

**Fig. 8:**
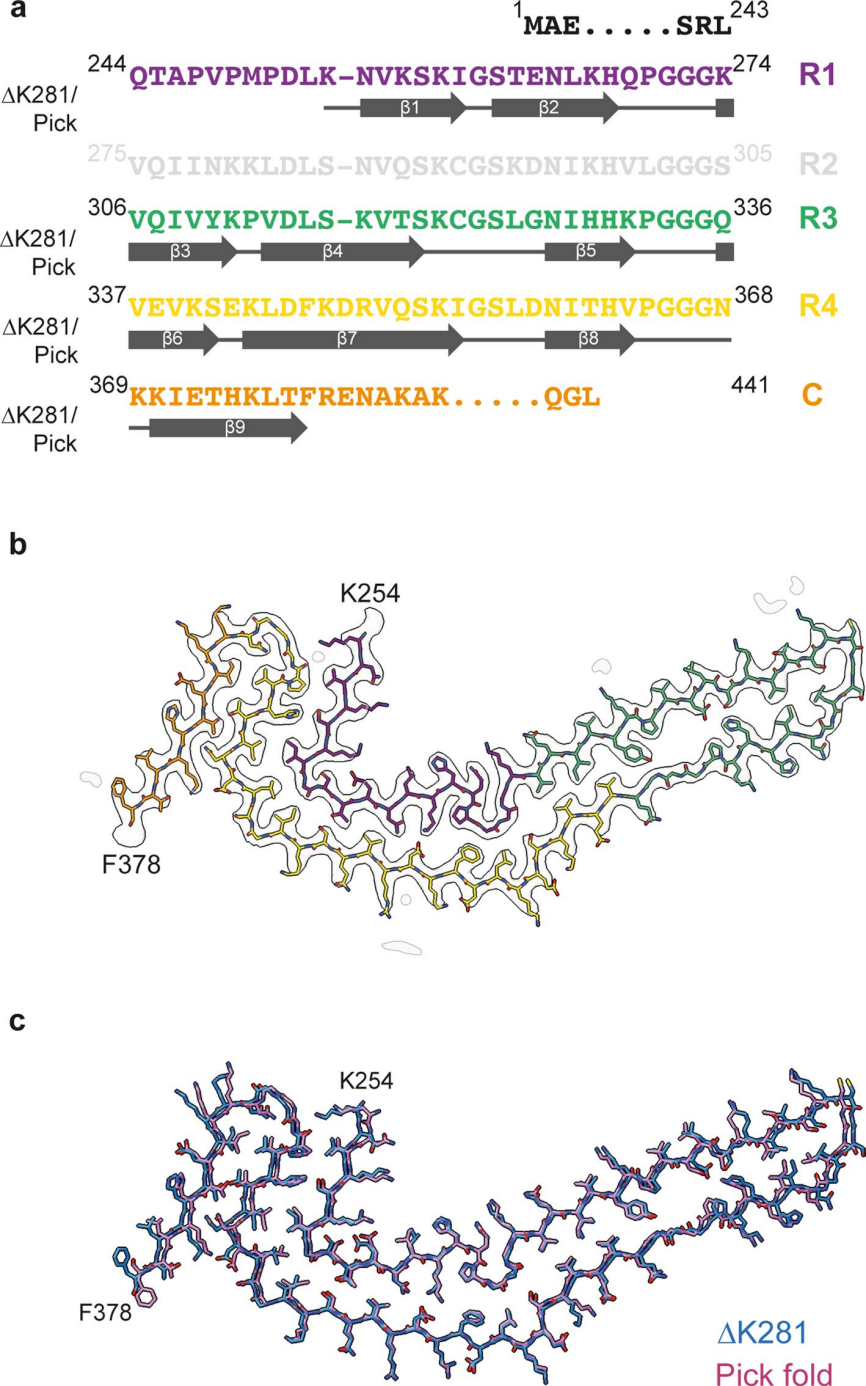
Tau filament fold from cases with *MAPT* mutation ∆K281. **a** Sequence alignment of the microtubule-binding repeats (R1-R4) of Tau with the observed 9 β-strand regions (arrows). **b** Sharpened high-resolution cryo-EM map of the ∆K281 singlet from grey matter of frontal cortex from case 2 with the atomic model overlaid. **c** Superposition of the backbone structures of the Tau filament fold from case 1 with mutation ∆K281 (blue) and the Pick fold (red)

We used cryo-EM to image Tau filaments that were extracted from temporal and frontal cortex of two cases with ∆K281 mutation. Filament structures were determined to a resolution of 2.6 Å, which was sufficient for atomic modelling (Figs. 7 and 8). The core of Tau filaments from cases with the ∆K281 mutation comprised the 21 C-terminal amino acids of R1, the whole of R3 and R4, as well as 10 amino acids after R4, but lacked residues V275-S305 of R2 (Fig. 8). The extended and two-layered filament core is identical to the Pick fold (13), comprising 9 β-strands. Identical filament folds were present in grey matter of temporal cortex from case 1 and grey and white matter of frontal cortex from case 2 (Fig. 7). More filaments were present in extracts from grey than white matter. Fewer than 10% of filaments from frontal cortex grey matter of case 2 were made of two identical protofilaments**.** However, the low resolution of the map prevented us from building an atomic model.

These findings were confirmed by immunogold negative stain electron microscopy, which showed that most filaments were identical to what we previously called NPFs, with a minority of WPFs (Supplementary Fig. 6). They had cross-over distances of 1,000 Å and widths of 50–150 Å (NPFs) and 50–300 Å (WPFs); they were decorated by anti-tau antibodies BR133, BR134, MC1 and AT8, indicating the presence of full-length, hyperphosphorylated tau. Filaments were not decorated by BR135, consistent with the presence of repeat 3 of Tau in the filament core.

## Discussion

Here we show that mutation ∆K281 in *MAPT* caused a familial form of FTD with focal atrophy of frontal and temporal lobes of the cerebral cortex, as well as abundant Pick bodies and glial Tau inclusions.

Like in Pick’s disease [10, 30], inclusions of assembled and hyperphosphorylated Tau were detected in nerve cells and glial cells by antibodies specific for 3R, but not 4R, Tau. Moreover, Bodian silver stained neuronal and glial inclusions, whilst no labelling was obtained with Gallyas–Braak silver or antibody 12E8, as shown previously for Pick’s disease [4, 37]. In agreement with previous findings [56], bTVBT4 stained the Tau inclusions of AD, but not of Pick’s disease. Inclusions from case 1 with mutation ∆K281 were also not labelled by bTVBT4. By contrast, compound HS-84 labelled the tau inclusions of AD, Pick’s disease and case 1 with mutation ∆K281. HS-84 interacts with regularly spaced lysine residues in grooves along amyloid filaments [27], whereas the most favourable ligand–protein interaction for bTVBT4 occurs at the hydrophobic pocket defined by I360, T361 and H362 in the Alzheimer Tau fold [16, 56]. Based on the cryo-EM structures, this pocket is not present in the Pick fold [13, 56]. It follows that the differential staining of Tau inclusions by HS-84 and bTVBT4 is in accordance with the different filament structures.

By immunoblotting of sarkosyl-insoluble fractions, strong bands of hyperphosphorylated Tau of 60 and 64 kDa bands and a weak band of 68 kDa were observed, consistent with the presence of 3R Tau. This agrees with previous findings in a case with mutation ∆K281 (called ∆K280) [58]. Antibody RD4 did not recognise recombinant Tau with mutation ∆K281. It also fails to label Tau when N279 is deamidated [8]. By contrast, anti-4R, which recognises Tau that is deamidated at N279, labelled both wild-type and mutant Tau. Using anti-4R, we failed to detect sarkosyl-insoluble Tau in temporal and frontal cortex of cases 1 and 2 by immunoblotting or Tau inclusions by immunohistochemistry.

There was thus no evidence to suggest that ∆K281 Tau was present in the inclusions, even though this mutation is known to increase the propensity of recombinant 4R Tau to assemble into filaments [2] and to reduce the ability of Tau to promote microtubule assembly [41]. The latter is consistent with K281 being one of three lysine residues that modulate Tau-microtubule interactions [23]. Acetylation of K280/281 has been reported to impair Tau-mediated stabilisation of microtubules and to enhance Tau assembly into filaments [57].

The pathogenic effects of the *MAPT* deletion mutation ∆K281 may be at the splicing level. It causes a shift in the 3R/4R Tau isoform ratio [11], the consequence of which is relative overexpression of wild-type 3R Tau and its assembly into filaments.

By cryo-EM, the structures of filaments extracted from grey matter of temporal cortex from case 1 and grey and white matter of the frontal lobe from case 2 with the ∆K281 mutation were identical to those from grey matter of frontal and temporal cortex of Pick’s disease. NPFs consisting of a single protofilament predominated. Protofilaments extended from residues K254 to F378 of 3R Tau. Unlike in other diseases with abundant Tau inclusions [46], TMEM106B filaments were not observed. We conclude that mutation ∆K281 in *MAPT* causes an inherited form of Pick’s disease, with neurons and glial cells containing filaments with the Pick fold of 3R Tau. These findings indicate that for the Pick fold, similar to what was observed for the AGD fold in cases with intron 10 mutations in *MAPT* [48], nerve cell and glial cell inclusions are made of identical tau folds. The presence of 4R Tau inclusions in glial cells in some cases of Pick’s disease [30] may indicate additional pathologies.

Other mutations in *MAPT* have also been reported to give rise to Pick-like disease, both clinically and neuropathologically [18]. However, none of them increased the 3R/4R Tau ratio. The characteristics of FTDP-17T caused by mutations G272V in exon 9 [5] and Q336H in exon 12 [54] come closest to sporadic Pick’s disease. However, Pick-like bodies in two G272V brains were Gallyas–Braak silver-positive. Moreover, some Pick-like bodies in Q336H brains were 4R tau- and Gallyas–Braak silver-positive.

Cases 1 and 2 were heterozygous for mutation ∆K281 and developed FTD. However, their parents, who were not genotyped, did not suffer from FTD. Similar findings have been reported in other cases with *MAPT* mutation ∆K281 [32, 41]. This apparent lack of family history raises the question of incomplete penetrance, similar to what has been suggested for *MAPT* mutation G389R in exon 13 [6, 53]. Germ-line mosaicism, which may have arisen during oogenesis or spermatogenesis, offers an alternative explanation. Germ-line mosaicism has previously been reported for *MAPT* mutation S305N in exon 10 [3].

FTLD-Tau includes Pick’s disease, PSP, GGT, CBD and AGD. We showed previously that these conditions are characterised by specific Tau filament folds [48]. It has been suggested that genetic forms of FTLD-Tau can also be described in this way and that the term FTDP-17 should be retired [17]. Cases with intron 10 mutations + 3 and + 16 in *MAPT* share a fold with AGD, suggesting that the relative overproduction of 4R Tau is sufficient to give rise to the AGD fold [48]. We now show that mutation ∆K281 in *MAPT* causes an inherited form of Pick’s disease, indicating that relative overproduction of 3R Tau is sufficient to give rise to the Pick fold. These findings establish that at least some cases of FTDP-17T are genetic forms of FTLD-Tau. It remains to be determined if this is also true of cases of FTLD that are caused by other *MAPT* mutations. Neuropathology at the atomic level, as determined by the cryo-EM structures of tau filaments, will be the deciding criterion.

## Supplementary Information

Below is the link to the electronic supplementary material.

Supplementary file1 (PDF 6884 KB)

## Data Availability

The cryo-EM map of the singlet filament (grey matter of frontal cortex from case 2) has been deposited in the Electron Microscopy Data Bank (EMDB) with accession number EMD-17383. The corresponding refined atomic model has been deposited in the Protein Data Bank (PDB) under accession number 8P34. Please address requests for materials to the corresponding authors.
