# Supplementary material for: Mutation ∆K281 in MAPT causes Pick’s disease
Source: Acta Neuropathol. 2023 Jun 23;146(2):211–26. doi: 10.1007/s00401-023-02598-6 (PMC10329087; doi:10.1007/s00401-023-02598-6)
Supplement: Supplementary file 1 — Supplementary file1 (PDF 6884 KB) [file 401_2023_2598_MOESM1_ESM.pdf]

MUTATION ΔK281 IN *MAPT* CAUSES PICK'S DISEASE

Manuel Schweighauser[1], Holly J. Garringer[2], Therése Klingstedt[1,3], K. Peter R. Nilsson[3], Masami Masuda-Suzukake[1,5], Jill R. Murrell[2,6], Shannon L. Risacher[4], Ruben Vidal[2], Sjors H.W. Scheres[1@], Michel Goedert[1@], Bernardino Ghetti[2@], Kathy L. Newell[2@]

[1] Medical Research Council Laboratory of Molecular Biology, Cambridge, UK

[2] Department of Pathology and Laboratory Medicine, Indiana University School of Medicine, Indianapolis, IN, USA

[3] Department of Physics, Chemistry and Biology, Lingköping University, Lingköping, Sweden

[4] Department of Radiology and Imaging Sciences, Indiana University School of Medicine, Indianapolis, IN, USA

Present addresses: [5]Department of Brain and Neuroscience, Tokyo Metropolitan Institute of Medical Science, Tokyo, Japan; [6]Department of Pathology and Laboratory Medicine, Children's Hospital of the University of Pennsylvania, Philadelphia, PA, USA

[@]Corresponding authors scheres@mrc-lmb.cam.ac.uk, mg@mrc-lmb.cam.ac.uk, bghetti@iupui.edu and klnewell@iu.edu

SUPPLEMENTARY TABLE

Table S1. Cryo-EM data acquisition and structure determination

	Case 1<br>Grey Matter<br>Temporal Cortex<br>Singlet	Case 2<br>Grey Matter<br>Frontal Cortex<br>Singlet<br>EMDB-17383<br>PDB 8P34	Case 2<br>Grey Matter<br>Frontal Cortex<br>Doublet	Case 2<br>White Matter<br>Frontal Cortex<br>Singlet
**Data collection and processing**				
Magnification	96,000	81,000	81,000	96,000
Voltage (kV)	300	300	300	300
Detector	Falcon 4	K3	K3	Falcon 4
Electron exposure (e–/Å^2)	30.0	37.7	37.7	30.0
Defocus range (μm)	1.5 to 2.5	1.8 to 2.8	1.8 to 2.8	1.5 to 2.5
Pixel size (Å)	0.824	0.93	0.93	0.824
Symmetry imposed	C1	C1	C2	C1
Initial particle images (no.)	126,179	1,000,880	67,253	144,637
Final particle images (no.)	9,865	258,445	67,253	33,927
Map resolution (Å)	3.42	2.61	4.9	3.15
FSC threshold = 0.143				
Helical rise (Å)	4.81	4.89	4.75	4.80
Helical twist (°)	-0.71	-0.73	-0.65	-0.72
**Refinement**				
Initial model used (PDB code)		6GX5		
Model resolution (Å)		2.56		
FSC threshold = 0.5				
Map sharpening *B* factor (Å^2)		-44.6		
Model composition				
Non-hydrogen atoms		2,920		
Protein residues		384		
Ligands		0		
B factors (Å^2)				
Protein		47.6		
R.m.s. deviations				
Bond lengths (Å)		0.006		
Bond angles (°)		1.284		
Validation				
MolProbity score		1.10		
Clashscore		0.69		
Poor rotamers (%)		0		
Ramachandran plot				
Favored (%)		94.57		
Allowed (%)		5.43		
Disallowed (%)		0		

SUPPLEMENTARY FIGURES

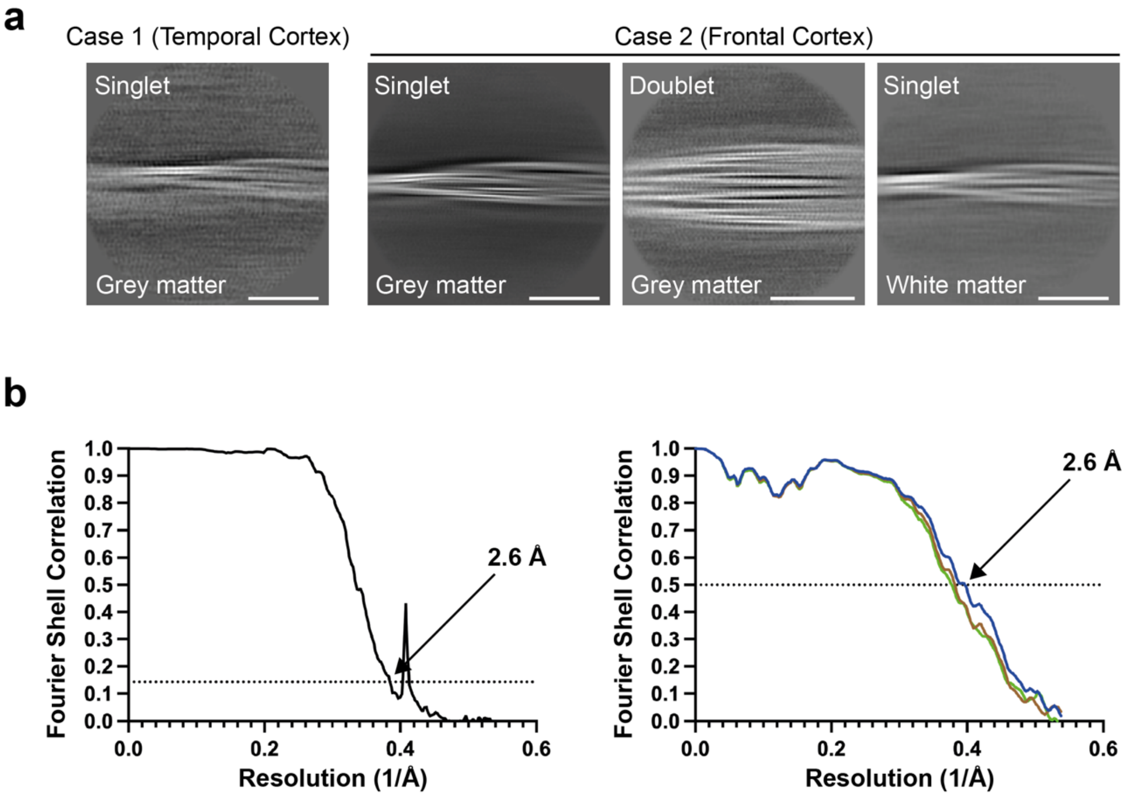

Figure S1. Cryo-EM 2D classifications and Fourier shell correlation (FSC) curves

(a), Representative 2D classification images of Tau filaments from cases 1 and 2. In addition to singlet filaments, for grey matter from case 2, a 2D class image of a doublet filament is also shown. Scale bar, 20 nm.

(b) Solvent-corrected FSC curve of cryo-EM half-maps (left panel) and model-to-map validation (right panel) for case 2 frontal cortex grey matter is shown. FSC curve between a model refined in half-map 1 and half-map 2 is shown in brown (model 1 vs. half-map 1) and FSC curve between the same model and half-map 2 is shown in green (model 1 vs. half-map 2).

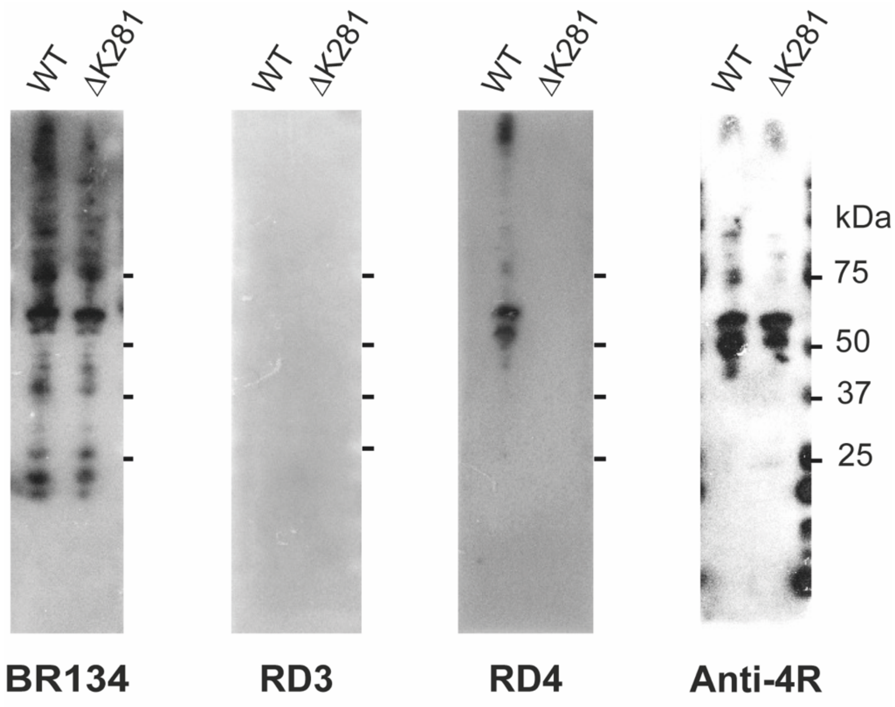

Figure S2. Immunoblot analysis of ΔK281 4R Tau

Immunoblotting of recombinant wild-type (WT) and ΔK281 1N4R Tau using anti-Tau antibodies BR134, RD3, RD4 and Anti-4R.

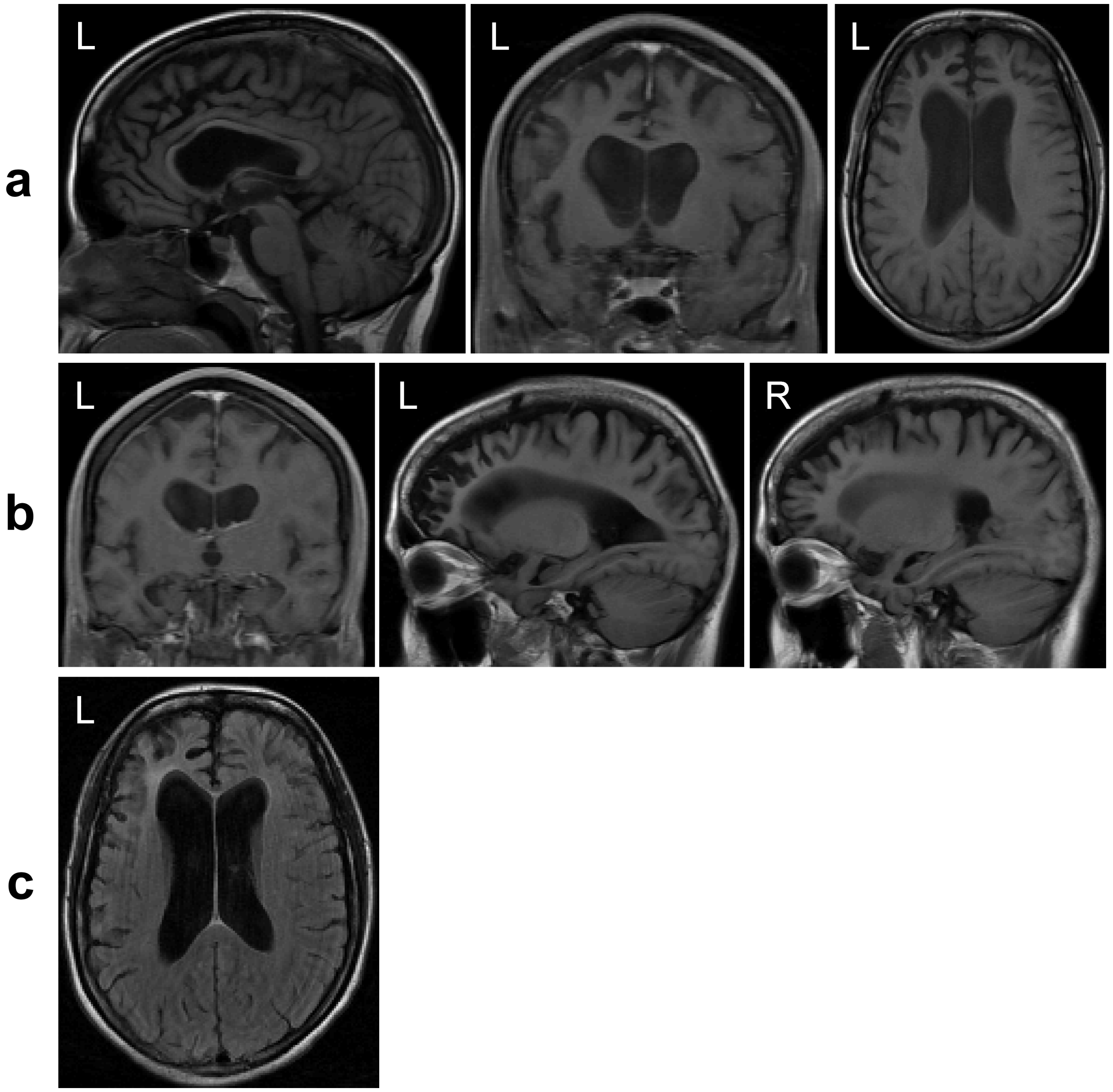

Figure S3. T1-weighted MRI scan of the brain of case 2 with *MAPT* mutation ΔK281

Scan obtained during the first visit, when case 2 was 47 years old.

(a,b,c), Sagittal, coronal and axial MR images show atrophy of the frontal lobe and narrowing of the gyri, severe atrophy of the corpus callosum and enlargement of the lateral ventricles.

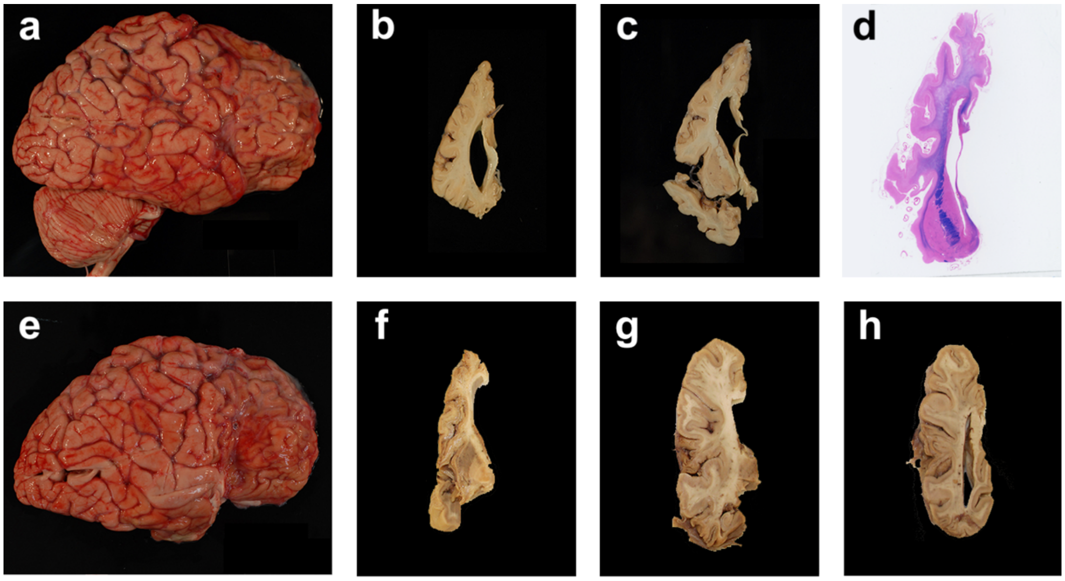

Figure S4. Lateral views and coronal sections of the brains of cases 1 and 2 with *MAPT* mutation ΔK281

(a,e), Lateral views of the fresh brains of cases 1 and 2 show marked atrophy of the frontal lobes; (b,c,f,g,h), Brain atrophy is more evident in fixed coronal slices; (d), Severity of neurodegeneration is shown in a coronal section stained with Luxol Fast Blue and haematoxylin-eosin. Note the atrophy of cortical gyri and corpus callosum, as well as the severe loss of myelin in white matter.

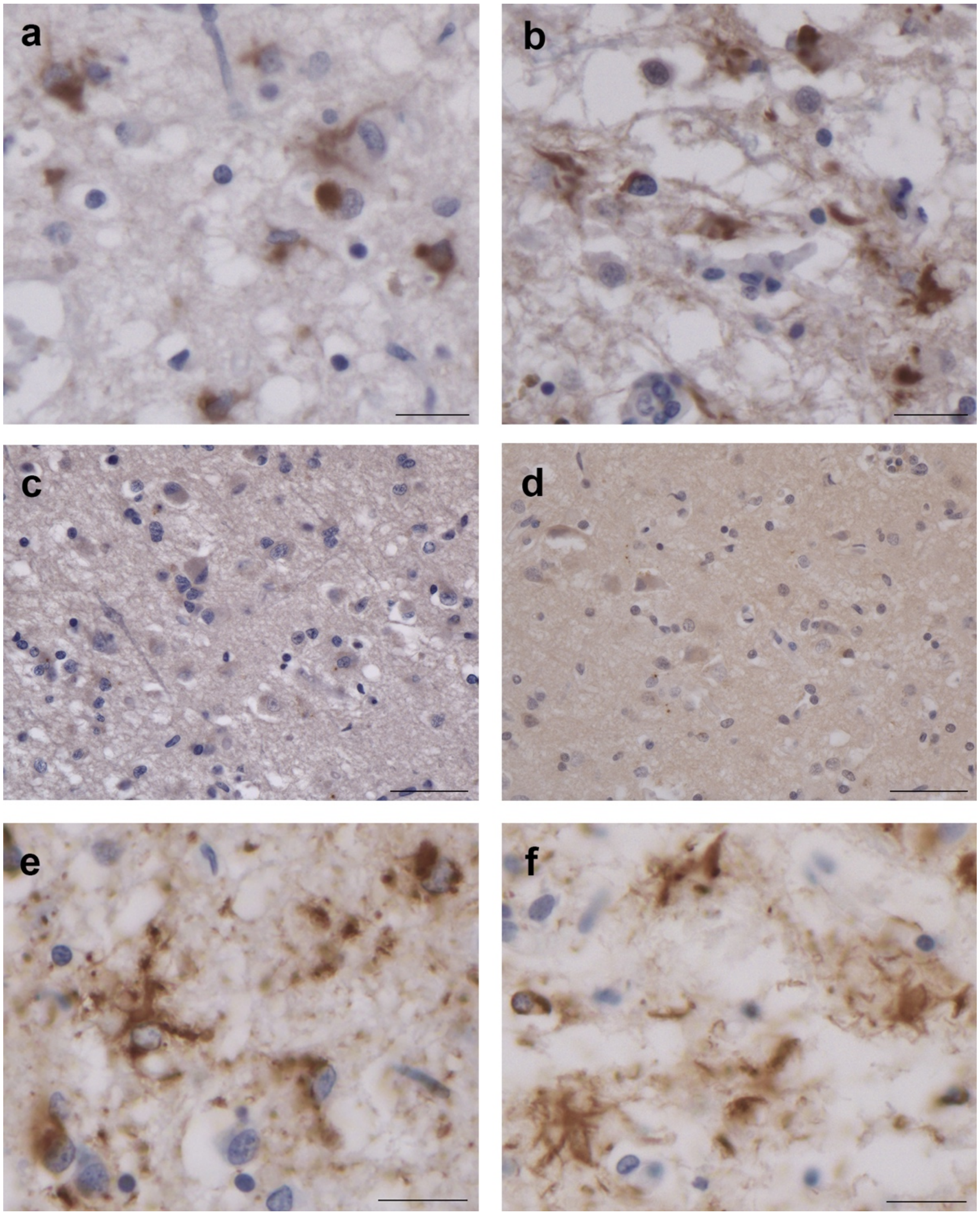

Figure S5. Ramified astrocytes in frontal cortex from cases 1 and 2 with *MAPT* mutation ΔK281

(a,b), RD3 tau-positive inclusions in ramified astrocytes from case 1 (a) and case 2 (b); (c,d), Anti-4R tau-negative inclusions in brain cells from case 1

(c) and case 2 (d); (e,f), AT8 tau-positive inclusions in ramified astrocytes from case 1 (e) and case 2 (f). Scale bars, 20 μm (a,b,e,f) and 50 μm (c,d).

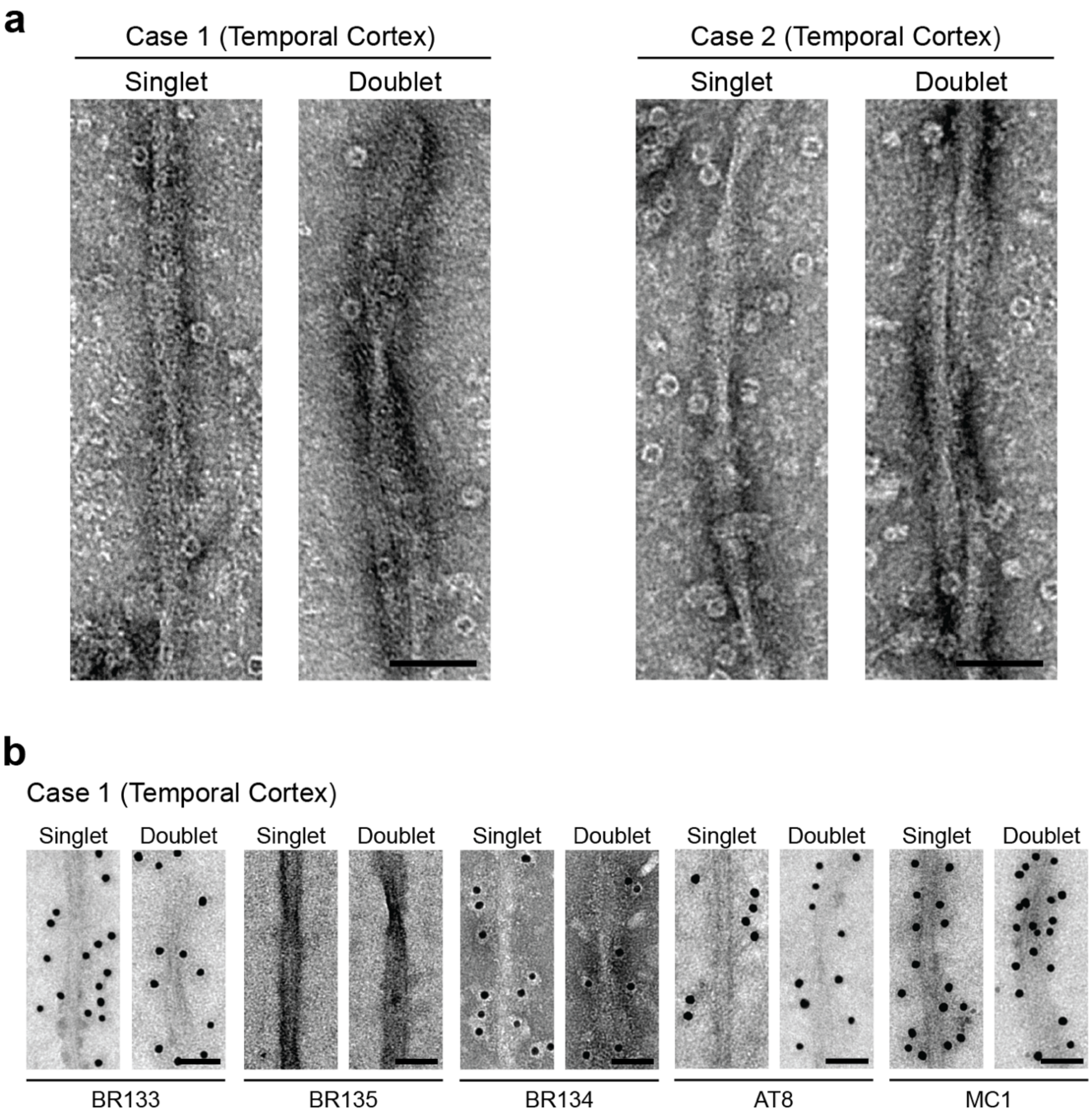

Figure S6. Negative stain immunoelectron microscopy of Tau filaments from grey matter of temporal cortex from cases 1 and 2 with *MAPT* mutation ∆K281

(a), Negative stain electron microscopy of singlet (NPFs) and doublet (WPFs) filaments from the sarkosyl-insoluble fraction of temporal cortex grey matter of cases 1 and 2 with the ∆K281 mutation. Scale bars, 50 nm.

(b), Immunogold negative stain electron microscopy of singlet and doublet Tau filaments from the sarkosyl-insoluble fraction of temporal cortex grey matter of case 1. Anti-Tau antibodies BR133, BR135, BR134, AT8 and MC1 were used. Scale bars, 50 nm.
